# Subacute thyroiditis following Mpox infection in a patient with human immunodeficiency virus

**DOI:** 10.1530/EDM-23-0034

**Published:** 2023-08-16

**Authors:** Emmanuel Ssemmondo, Mohamed Akasha Idris, Damian Mawer, Nicholas Easom, Jonathan Thow

**Affiliations:** 1 Academic Diabetes, Endocrinology & Metabolism, University of Hull, Hull, United Kingdom; 2 Hull University Teaching Hospital NHS Trust, Hull, United Kingdom; 3 York and Scarborough Teaching Hospitals NHS Foundation Trust, Hull, United Kingdom

**Keywords:** Adult, Male, White, United Kingdom, Thyroid, Thyroid, Infectious diseases, New disease or syndrome: presentations/diagnosis/management, August, 2023

## Abstract

**Summary:**

Mpox (MPX) formerly known as monkeypox was declared a public health emergency of international concern, following an outbreak that commenced in May 2022. We report a case of subacute thyroiditis following MPX infection. To our knowledge, it is the first documented incidence of this complication in humans. A 51-year-old male, with a well-controlled human immunodeficiency virus (HIV) infection on antiretroviral therapy, was reviewed 3 weeks after a positive test for MPX. The acute skin lesions and initial systemic symptoms had resolved, but he described significant neck discomfort, fatigue, weight loss and night sweats. Blood tests showed a raised C-reactive protein, free T4 and suppressed thyroid-stimulating hormone. His thyroid antibodies were negative. He was treated initially with carbimazole and propranolol, pending exclusion of any other intercurrent infection. A chest radiograph was normal; blood cultures and a combined nose and throat swab for respiratory virus PCR testing were negative. Following this, he commenced a 2-week course of prednisolone; his symptoms resolved completely within 24 h of starting. He subsequently developed hypothyroidism, which was treated with levothyroxine. The clinical features, abnormal thyroid function, raised CRP and negative thyroid antibodies 3 weeks post-MPX positive test was consistent with viral subacute thyroiditis. This case demonstrates that, as described following other viral infections, MPX can cause subacute thyroiditis, which follows a similar course to the classic form of subacute thyroiditis. Clinicians should be aware of this potential endocrine complication when attending to patients with MPX.

**Learning points:**

- Subacute thyroiditis can present following mpox virus infection.
- Its course is similar to the classic form of subacute thyroiditis and steroids are effective.
- It is important to exclude other concurrent infections prior to starting steroids, especially for patients who are immunosuppressed or in other high-risk groups.

## Background

Mpox (MPX) virus, previously known as monkeypox, is a double-stranded DNA virus belonging to the family Poxviridae (1). It is endemic in west and central Africa. In May 2022, mpox cases were reported in the United Kingdom, Portugal and Italy, mostly among men who have sex with men (MSM). This marked the start of a global outbreak that was declared a public health emergency of international concern by the World Health Organization (WHO) 2 months later (1).

Human-to-human transmission of MPX is by contact with body fluids and skin lesions of infected individuals. The three phases of MPX infection described are the incubation, prodrome and eruptive (2). Following incubation (mean period of about 9 days for the current outbreak), the prodromal phase includes fever, headache, fatigue and arthralgia. The eruptive phase is characterised by skin lesions. These classically pass through macular, papular and vesiculo-pustular stages, before crusting over. In one analysis, 98% of patients had a rash during the eruptive phase, with anogenital skin and mucosal lesions reported in 71% of patients (1).

Thyroiditis has been reported in association with MPX infection in animal experiments (3). The current MPX outbreak has led to calls to consider the possibility of thyroid illness in humans infected with the virus (4).

We present a case of MPX subacute thyroiditis in a male patient with well-controlled HIV infection. To the best of our knowledge, this is the first case of subacute thyroiditis following MPX infection reported in the literature.

## Case presentation

**Figure 1:**
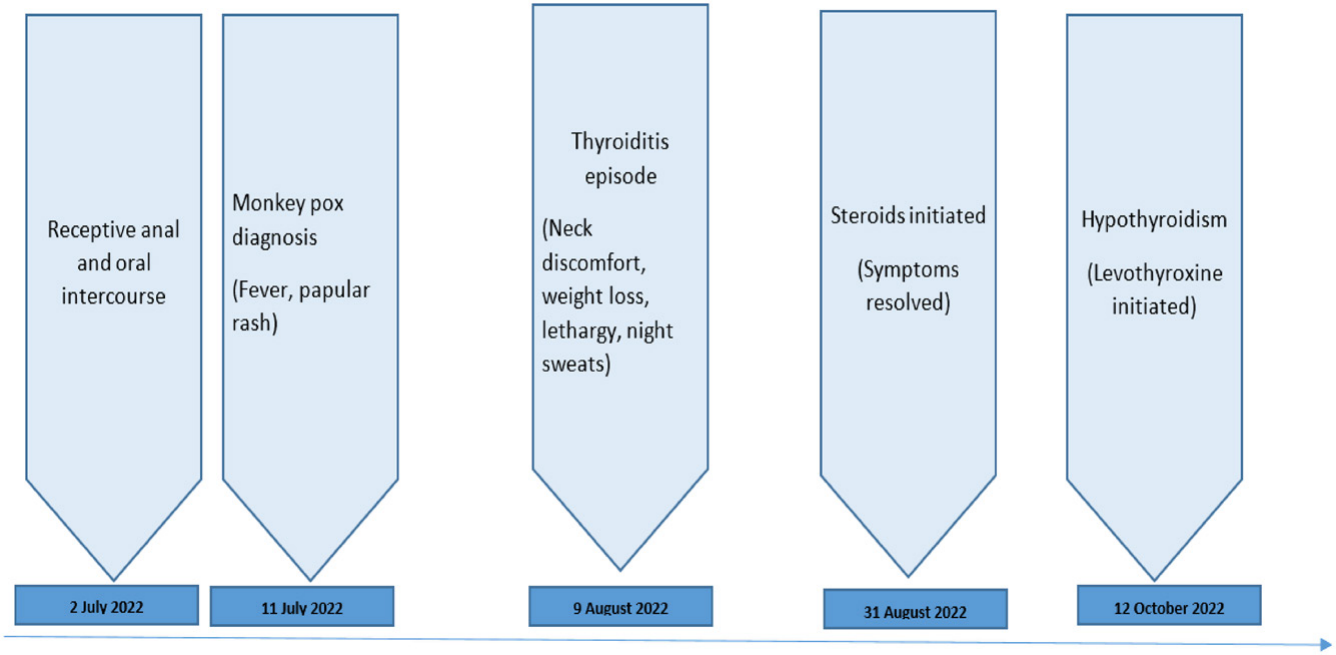
Time line showing highlights of the disease progression.

A 51-year-old Caucasian male presented to the genitourinary medicine clinic, with a crusted perioral lesion and scattered papular lesions on the limbs. These followed a 3-day prodromal illness with fever, generalised myalgias and headache. The illness began 5 days after unprotected anal and oral intercourse with a casual male partner (see timeline in Fig. 1). A swab of the perioral lesion confirmed MPX infection (see below). His symptoms and skin lesions all resolved within 10 days. Three weeks later, he presented to the same clinic with weight loss, fatigue, night sweats and intermittent shivering. He also reported neck discomfort.

The patient was diagnosed HIV positive in 2006. The infection is well controlled on antiretroviral therapy, started in 2008. Other past medical history included HIV-related peripheral neuropathy, recurrent genital herpes, hypercholesterolaemia and non-alcoholic fatty liver disease. His medications were tenofovir disoproxil, emtricitabine, raltegravir, rabeprazole, acyclovir and atorvastatin. On examination, he had a modest goitre, which was moderately tender. There was no regional lymphadenopathy. His cardiovascular and respiratory examinations were normal.

## Investigations

MPX infection was diagnosed from a positive PCR test done on a dry swab from the perioral lesion. UK Health Security Agency’s (UKHSA) Rare and Important Pathogens Laboratory (RIPL) in Salisbury, UK, performed the test. Due to infection control concerns, blood tests were not performed until the MPX infection had resolved and the patient re-presented with thyroiditis symptoms. Results are shown in Table 1. The erythrocyte sedimentation rate (ESR) was raised at 59 with a C-reactive protein (CRP) of 51 mg/L (normal range (NR): 0–5). Thyroid function tests (TFTs) showed a suppressed serum thyroid-stimulating hormone (TSH) level of <0.01 Mu/L (NR: 0.27–4.2), serum-free T4 of 34 pmol/L (NR: 11–22) and free T3 of 9.1 pmol/L (NR: 3.1–6.8). Anti-thyroid antibodies were negative (TSH receptor antibodies (TRAb): 0.9 U/L, NR: <1.8; thyroid peroxidase antibodies (TPO): <1 U/mL, NR: 0–75). Based on the abnormal TFT results, the patient was referred to an endocrinology clinic, where a diagnosis of viral thyroiditis was made, because of the clinical picture and blood results. Due to his febrile symptoms, the patient was then transferred to the Regional Infectious Diseases Unit for assessment and exclusion of an intercurrent infection prior to starting steroids. Investigations performed there included a nasopharyngeal swab for respiratory viruses (severe acute respiratory syndrome coronavirus-2 (SARS-COV-2), adenovirus, human metapneumovirus, rhinovirus, enterovirus, influenza A and B, parainfluenza virus 1–4, and respiratory syncytial virus, *Bordetella parapertussis*, *Chlamydia pneumonia* and *Mycoplasma*), blood cultures and a chest radiograph. These were all negative. Mpox PCR of throat swab, whole blood and semen were negative at this stage. An HIV viral load was undetectable and the CD4 count was 994 cells/mm^3^ (NR: 300–1400 cells/mm^3^).

**Table 1:** Blood results at the initial endocrine clinic review.

Test	Normal Range	Result
CRP, mg/L	0–5	51
Haemoglobin, g/L	130–180	122
White cell count, ×10^9^/L	4.0–11.0	9.9
Neutrophils, ×10^9^/L	2.0–8.0	6.3
Lymphocytes, ×10^9^/L	0.5–4.5	2.5
Platelets, ×10^9^/L	150–450	406
TPO antibodies, U/ml	0–75	<1
Sodium, mmol/L	133–146	144
Potassium, mol/L	3.5–5.3	4.1
Urea, mmol/L	2.5–7.8	5.7
Creatinine, µmol/L	59–104	66
Calcium, mmol/L	2.20–2.60	2.31
Adjusted calcium, mmol/L	2.20–2.60	2.37
Magnesium, mmol/L	0.70–1.00	0.92
Inorganic phosphate, mmol/L	0.80–1.50	1.27
Random cortisol, nmol/L	133–537*	187
Total protein, g/L	60–80	70
ALP, IU/L	30–130	130
ALT level, U/L	0–45	20
Total bilirubin, µmol/L	<21	9
CD4 count, cells/mm^3^	300–1400	994

*6:00–10:00 am values ALP, alkaline phosphatase; ALT, alanine transaminase; TPO, thyroid peroxidase.

## Treatment

No specific treatment was given for the MPX infection. Following the abnormal TFTs, the patient was started on oral propranolol 10 mg three times a day and carbimazole 20 mg once a day. These were continued until an intercurrent infection had been adequately excluded, at which point the patient was given oral prednisolone 20 mg once a day for 1 week.

## Outcome and follow-up

The patient was reviewed 7 days after starting prednisolone. He reported that his symptoms had resolved within a day of starting the steroids. The carbimazole was therefore stopped at this visit and the propranolol was stopped after a 3-day tapering course. The prednisolone dose was reduced to 10 mg once a day for another week and then rapidly tapered off. The TFTs during the follow-up period are shown in Table 2.

**Table 2:** Thyroid function test results during the follow-up period.

Test	Reference	Follow-up					
		05.01.21	09.08.22	23.08.22	06.09.22	11.10.22	01.11.22
Free T4 (pmol/L)	11–22	17	34	46	24	4	16
Free T3 (pmol/L)	3.1–6.8	–	9.1	10.2	5.0	2.9	–
TSH (mU/L)	0.27–4.2	1.5	<0.01	<0.01	<0.01	64.1	3.5

Six weeks after treatment with steroids, the TFT results showed hypothyroidism. He was then started on levothyroxine 100 µg once a day. Three weeks after starting levothyroxine, the results had improved (see Table 2). He remains clinically euthyroid and under endocrinology follow-up at the time of writing.

## Discussion

We describe an episode of subacute thyroiditis following infection with MPX virus in a patient with HIV infection. To the best of our knowledge, this is the first such case reported in humans. The diagnosis of thyroiditis was based on clinical features of thyrotoxicosis, a moderately tender goitre, a suppressed TSH in the context of elevated free T3 and T4 levels, raised CRP and negative anti-thyroid antibodies. In addition, the symptoms resolved after treatment with prednisolone. The infectious diseases specialists excluded other infections. His HIV infection was well controlled, which strongly suggests that MPX was responsible for subacute thyroiditis.

Like most cases of subacute thyroiditis, our patient presented with features of thyrotoxicosis. His illness followed the classical course of hyperthyroidism, followed by hypothyroidism. He remains under endocrinology follow-up. It is likely that his thyroid function will normalise, although 5% of cases remain hypothyroid permanently (5).

There was no ultrasound scan of the thyroid done, which is a limitation of our case report, since some authors have identified it as one of the main criteria in making a diagnosis of subacute thyroiditis (6). Ultrasound of the thyroid can play a significant role in not only diagnosis but also monitoring of subacute thyroiditis along with biochemical thyroid function tests (6). In our patient, however, an ultrasound of the thyroid was not done due to the limited time the team had. The patient was transferred to the regional Infectious diseases centre as a priority to exclude an intercurrent infection. The ultrasound scan had not been done by the time the transfer took place. Once the intercurrent infection had been ruled out, due to the strong circumstantial evidence of thyroiditis, the patient was given steroids without an ultrasound scan of the thyroid.

Subacute thyroiditis has been described following several viral infections (7). Mumps, enteroviruses (such as Coxsackie viruses), influenza, Epstein-Barr virus and cytomegalovirus are among the viruses that have been implicated in the past. SARS-CoV-2, the cause of COVID-19 infection, has also been reported to cause subacute thyroiditis (8). The literature on the effect of the MPX virus on the thyroid gland is scarce. A study in animal models found thyroiditis as one of the effects of the MPX virus on the endocrine system (3). Our patient presented with thyrotoxicosis symptoms 3 weeks following the initial diagnosis of MPX infection. The mechanism by which the MPX virus causes thyroiditis has not been previously described and therefore remains not fully understood. It is thought to involve a combination of direct viral invasion and/or immune-mediated inflammation resulting in thyroid tissue damage. When the thyroid follicles are infiltrated, there is disruption of the basement membrane, which causes the follicles to rapture (7, 9). Findings from a histologic and immunochemical study suggest that cellular immune response could be important in the pathogenesis of subacute thyroiditis (9). In this mechanism, the injury to the thyroid gland occurs when the cytolytic T cells recognize the viral and cell antigens present in a complex (9). This results in an inflammatory destruction of the thyroid follicles (10).

As the number of infections in the current global outbreak of MPX infection grows, it is likely that other cases of subacute thyroiditis will occur. Clinicians looking after such patients should be alert to this possibility.

## Declaration of interest

The authors declare that there is no conflict of interest that could be perceived as prejudicing the impartiality of the research reported.

## Funding

This research did not receive any specific grant from any funding agency in the public, commercial or not-for-profit sector.

## Patient consent

Written informed consent for publication of their clinical details was obtained from the patient.

## Patient’s perspective

I am very grateful for the care and tenacity of my HIV consultant Dr Mawer, who patiently tested and retested me until the issues with my thyroid were detected. My symptoms were very specific and very vague. I have a very good and longstanding doctor–patient relationship, and it was largely due to this that I was able to go into the weirder and more obscure symptoms (like a feeling of sunburn on my face and head) and very low mood and lethargy, without him going off at a misleading tangent. I am also very grateful to the thyroid department at York hospital and to the clinical nurse specialist Stuart Cowley and Dr Thow. They both supported me through an extremely difficult time. My symptoms were very strange, not that typical for thyroiditis and I know were very concerning for Dr Thow, hence my stay at the infectious diseases unit, but it turned out to be, as suspected, thyroiditis. I’m just very glad that everyone took great pains to make sure that any other possibilities were ruled out and I was given swift and effective treatment.

## Author contribution statement

Concept: ES and JT. Data collection and processing: ES, MAI, DM and JT. Writing: ES, MAI, DM and NE. Analysis and interpretations: ES, MAI, DM, NE and JT. Literature search: ES, MAI, DM, NE and JT.
